# The relationship between platelet count and postoperative acute kidney injury and long-term prognosis in neurosurgical critically ill patients: A retrospective study

**DOI:** 10.1371/journal.pone.0343653

**Published:** 2026-02-27

**Authors:** Shu Yang, Shuo Zhang, Guoqing Li, Guowei Zhu, Linfeng Fang, Minmin Zhu

**Affiliations:** 1 Wuxi School of Medicine, Jiangnan University, Wuxi, China; 2 Department of Anesthesiology and Pain Medicine, Wuxi No.2 People's Hospital (Jiangnan University Medical Center), Wuxi, China; 3 The Second Hospital of Tianjin Medical University, Tianjin, China; Sai Gosavi Specialty Clinic / Nano Hospitals Bangalore / Saraswati Specialty Clinic, INDIA

## Abstract

**Objective:**

This study was designed to investigate the associations among platelet (PLT) count upon admission to the intensive care unit (ICU), postoperative acute kidney injury (AKI), and long-term prognosis (one-year mortality risk) in patients undergoing neurosurgical operations.

**Methods:**

This study conducted a retrospective analysis based on the MIMIC-IV database, including patients who underwent neurosurgery and were admitted to the ICU. Platelet count information at admission was collected. The primary endpoint were AKI within 7 days of ICU admission and mortality within one year after surgery. For the primary endpoint, a multivariate logistic regression model combined with restricted cubic spline was used for statistical analysis to explore the potential association between platelet count and AKI, and subgroup analysis was conducted to assess the stability of the results. For the other endpoint, a restricted cubic spline was used to construct a visualization relationship graph, and a Cox multivariate regression model was further established and a cumulative mortality curve was drawn. Sensitivity analyses were performed using both the first recorded platelet count following ICU admission and the 24-hour mean platelet count to assess the robustness of our findings. Additionally, an independent validation cohort was constructed using the MIMIC-III database to conduct external validation.

**Results:**

A total of 1605 patients were included in this study, with a median age of 60 years, among whom 875 were male (54.5%). Among the 1605 patients, 607 (37.82%) developed acute kidney injury within 7 days of hospitalization. Logistic regression analysis showed that compared with patients in the first quartile of platelet count (Q1 ≤ 179), those in the third quartile (Q3 > 234) had a significantly lower risk of developing acute kidney injury within 7 days of hospitalization (Model 1:HR = 0.42, 95% CI: 0.33–0.54; Model 2: HR = 0.67, 95% CI: 0.59–0.76; Model 3: HR = 0.57, 95% CI: 0.43–0.74; Model 4: HR = 0.62, 95%CI: 0.47–082; all P-values < 0.050). Furthermore, when the platelet count is below 204 × 10⁹/L, the risk of AKI occurrence in patients increases significantly. Cox multivariate regression analysis of the secondary endpoint showed that both relatively low platelet count (≤ 179) and relatively high platelet count (> 234) were associated with an increased risk of death within 1 year, with the former association being particularly significant (HR = 1.53, 95% CI: 1.17–2, P = 0.002). Sensitivity analyses yielded directionally consistent results. In the MIMIC-III external validation cohort, the associations between platelet levels and risks of AKI as well as long-term mortality remained generally consistent.

**Conclusion:**

In neurosurgical patients admitted to the ICU, early platelet levels within the first 24 hours were associated with the incidence of AKI within 7 days and long-term outcomes. A lower platelet count during the early ICU period was associated with an increased risk of AKI and poorer prognosis. This association appeared to be non-linear, the range of 179–234 × 10⁹/L corresponded to a lower risk or better prognosis. Platelet count can be a potential tool for risk stratification, but it is not sufficient to support clinical intervention decisions based on causal relationships.

## 1. Introduction

Neurosurgical operations, including intracranial tumor resection, aneurysm clipping, or treatment of traumatic brain injury (TBI), are frequently accompanied by a substantial risk of perioperative hemorrhage and hemodynamic instability. This situation is prone to triggering a series of postoperative complications [1,2]. Among these complications, acute kidney injury (AKI) is highly prevalent. It is often induced by ischemia, inflammation, or exposure to nephrotoxic drugs. AKI affects 67% of patients in the intensive care unit (ICU) and is directly associated with an in-hospital mortality rate of 40–50% [3–5].

Although existing research has delved deeply into the roles of intraoperative blood loss, hypotension, and nephrotoxic drugs in the development of AKI, the relationship between postoperative platelet (PLT) abnormalities and AKI remains ambiguous [6,7]. Particularly for neurosurgical patients, surgical trauma, the need for blood transfusion, and coagulation disorders commonly result in alterations in both the quantity and function of platelets. These changes may, in turn, impact renal function through mechanisms such as microcirculation disturbances and inflammatory responses [1].

Platelets are not only involved in the hemostatic process but also play a pivotal role in inflammatory responses and endothelial function [8]. Thrombocytopenia can exacerbate ischemia-reperfusion injury. Moreover, abnormal platelet function or a low platelet count may lead to the formation of microthrombi in renal tubules and an enhancement of oxidative stress, thus promoting kidney injury [9,10]. Clinical studies have shown that among critically ill patients suffering from infectious endocarditis, the platelet count can independently predict the risk of mortality within 28 days [11]. Moreover, existing research on sepsis indicates that there exists a crucial threshold for platelet count (176 × 10⁹/L) in affected patients. Mortality risk increases when the platelet count is either below or above this threshold [12].

Neurosurgical patients present with clinical features such as surgical bleeding and the use of mannitol, all of which can cause changes in platelet quantity or function. However, the association between these factors and AKI lacks substantial large-scale evidence. Although dynamic changes in platelet counts among ICU patients have been shown to be associated with short-term adverse events, there is still a lack of direct evidence regarding whether they independently influence the risk of AKI and long-term survival rates in the neurosurgical postoperative population [9,13].

Most existing AKI prediction models incorporate parameters such as serum creatinine and urine output. However, platelets, as a readily available parameter in routine ICU tests, have not had their prognostic value fully exploited [14,15]. More significantly, there is currently no consensus on the intervention threshold for platelet abnormalities. For example, it remains unclear whether platelet transfusion is necessary to prevent AKI and improve the prognosis of high-risk individuals. This lack of consensus poses challenges to clinical decision-making, especially for neurosurgical patients who are at risk of both bleeding and AKI. Therefore, investigating the correlation between platelet levels upon ICU admission and AKI, as well as long-term prognosis, in neurosurgical patients is of great significance for optimizing risk stratification and enhancing clinical outcomes.

This study is designed to assess the relationship between platelet concentrations and the likelihood of developing AKI within seven days among neurosurgical patients during their ICU stay. Simultaneously, it aims to elucidate the relationship between platelet levels and the 1-year mortality rate in this patient group and identify the possible optimal platelet range for such patients.

## 2. Materials and methods

### 2.1. Data origin

The dataset utilized in this research was obtained from the MIMIC-IV database (version 3.1). All personally identifiable information related to patients in the database has been removed to ensure confidentiality. As a result, the study was granted exemption from requiring ethical review and informed consent. The first author of this paper, SY, had complete access to the MIMIC-IV database and conducted the retrieval of pertinent data (access credential ID: 62274870). The data were accessed for research purposes on 25 May 2024. The original data collection from source institutions (e.g., Beth Israel Deaconess Medical Center) was approved by their respective Institutional Review Boards (IRBs) with waived informed consent due to the retrospective and anonymized nature of the data. In the MIMIC-IV database, patients’ private information is encrypted. According to national legislation and institutional requirements, this study does not require ethical approval or informed consent.

### 2.2. Study population

**Fig 1:**
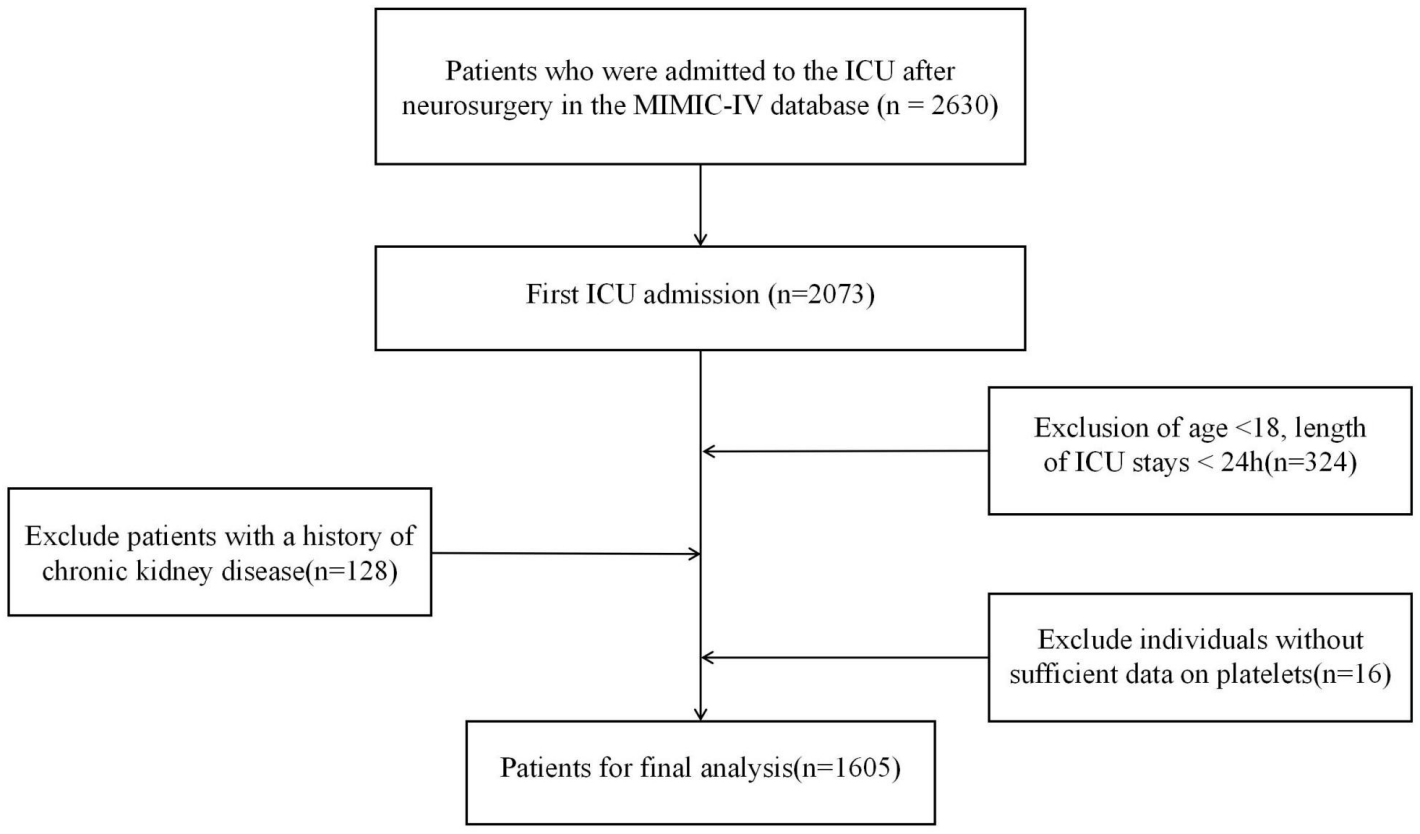
Flow diagram of inclusion and exclusion of the study population.

Inclusion criteria: Patients who were first admitted to the intensive care unit (ICU) after neurosurgical procedures. Exclusion criteria: (1) Patients aged less than 18 years old; (2) Patients with an ICU length of stay less than 24 hours; (3) Patients lacking platelet data within 24 hours of admission; (4) Patients with a history of chronic kidney disease. AKI was defined in accordance with the Kidney Disease: Improving Global Outcomes (KDIGO) criteria, incorporating both serum creatinine levels and urine output measurements. Creatinine-based staging was determined using measured serum creatinine values: baseline creatinine was defined as the lowest serum creatinine level within the 7 days prior to admission, while the 48-hour criterion was assessed using the lowest serum creatinine level within the 48 hours prior to admission. Urine output-based staging was defined based on KDIGO thresholds: < 0.5 mL/kg/h for 6 consecutive hours, < 0.3 mL/kg/h for 12 consecutive hours, or anuria for 12 consecutive hours. The final AKI stage at each time point was determined as the highest stage derived from creatinine-based and urine output-based staging. And AKI events were assessed within the first 7 days following ICU admission. As presented in **Fig 1**, after the application of these criteria, a total of 1,605 patients were enrolled in this study.

### 2.3. Data extraction

The research collected and evaluated the following information: (1) Patient demographics, including age, gender, ethnicity, duration of hospitalization, and ICU admission period; (2) Physiological parameters such as heart rate, systolic and diastolic blood pressure (SBP,DBP), mean arterial pressure (MAP), respiratory rate, body temperature, and blood oxygen saturation (SpO2); (3) Laboratory test results encompassing hematocrit, hemoglobin levels, platelet count, white blood cell count (WBC), blood urea nitrogen (BUN), creatinine concentration, anion gap, sodium, potassium, calcium, chloride, bicarbonate, international normalized ratio (INR), prothrombin time (PT), and partial thromboplastin time (PTT); (4) Existing comorbid conditions, such as myocardial infarction, congestive heart failure, peripheral vascular disease, dementia, cerebrovascular disorders, chronic obstructive pulmonary disease (COPD), rheumatologic diseases, peptic ulcer, mild to severe liver dysfunction, diabetes with and without complications, paraplegia, malignancies, metastatic cancers, and AIDS; (5) Clinical severity scores, including the Charlson Comorbidity Index, Acute Physiology Score III (APSIII), Simplified Acute Physiology Score II (SAPSII), and Oxford Acute Severity of Illness Score (OASIS); Additionally, recorded interventions included the use of mechanical ventilation and continuous renal replacement therapy (CRRT).We also extracted data on mannitol administration, blood transfusion exposure, perioperative hypotension(early ICU 24h hypotension as a proxy for perioperative hypotension), and antiplatelet medication use within 24 hours after patients were admitted to the ICU postoperatively. The lowest platelet count selected within the first 24 hours after admission to the ICU was chosen as the main exposure factor, as it is considered a sensitive marker of early platelet consumption and systemic stress in critically ill patients. Studies have shown that the lowest platelet count is more closely associated with organ dysfunction and adverse outcomes [16].

### 2.4. Research outcomes

The primary endpoints of this study were the incidence of acute kidney injury within 7 days after admission to the ICU and the one-year mortality rate.

### 2.5. Statistical analysis

Patients were categorized into three groups according to the tertiles of their platelet counts: the Q1 group (PLT ≤ 179, n = 524), the Q2 group (179 < PLT ≤ 234, n = 541), and the Q3 group (PLT > 234, n = 540). In accordance with clinical significance and relevant literature, we conducted our analysis using the minimum platelet count measured within 24 hours of a patient's initial ICU admission. The missing values for all study variables were less than 10%. For these missing values, we employed multiple imputation for data filling. Baseline features were described using descriptive statistics, expressed as n (%). Intergroup differences were assessed through chi-square tests or Fisher's exact tests. Continuous variables with a normal distribution were presented as mean ± standard deviation, and group comparisons were conducted using independent sample t-tests. For variables that did not follow a normal distribution, results were reported as median (interquartile range: Q1, Q3), and differences were analyzed using the Wilcoxon rank-sum test. The occurrence of acute kidney injury was evaluated via univariate and multivariate logistic regression analyses. To assess prognosis, multivariate Cox regression models were employed, and Kaplan-Meier survival curves were constructed to compare survival rates among patients with varying platelet levels. Furthermore, restricted cubic spline (RCS) techniques were applied to explore the association between platelet levels and the two studied outcomes, and forest plots were utilized to investigate potential interactions. To assess the robustness of our findings, we conducted sensitivity analyses by replacing the primary exposure variable (minimum platelet count within the first 24 hours of ICU admission) with the first measured platelet count post-ICU admission and the mean platelet count during this period. All analyses employed the same multivariate logistic/Cox regression model with restricted cubic splines incorporated. The statistical analysis was performed with R software and Free Statistics software. A p-value below 0.05 was regarded as statistically significant.

## 3. Results

### 3.1. Analysis of the Incidence of AKI

#### 3.1.1. Population baseline.

In comparison to patients with normal kidney function (NKD), those with AKI exhibited a greater male predominance, were of more advanced age, and demonstrated elevated heart rate, systolic blood pressure, respiratory rate, and SpO2 levels. Additionally, AKI patients showed higher levels of blood glucose, albumin, BUN, serum creatinine, sodium, INR, PT, and PTT. They also presented with greater disease severity and experienced longer durations of hospitalization and ICU admission. Furthermore, baseline platelet counts were notably lower in AKI patients when compared to their NKD counterparts (P < 0.001, **Table 1**).

**Table 1:** Baseline characteristics of patients in the NKD group and AKI group.

Variables	Total (n = 1605)	NKD (n = 998)	AKI (n = 607)	*p*-value
**Characteristics**				
Gender, n (%)				< 0.001
Female	730 (45.5)	490 (49.1)	240 (39.5)	
Male	875 (54.5)	508 (50.9)	367 (60.5)	
Age (years), n (%)				< 0.001
<65	990 (61.7)	654 (65.5)	336 (55.4)	
>=65	615 (38.3)	344 (34.5)	271 (44.6)	
race, n (%)				0.028
White	1071 (66.7)	690 (69.1)	381 (62.8)	
African American	131 (8.2)	77 (7.7)	54 (8.9)	
Asain	61 (3.8)	40 (4)	21 (3.5)	
Other	342 (21.3)	191 (19.1)	151 (24.9)	
Heart rate (beats/min), Mean ± SD	81.4 ± 13.9	80.2 ± 13.5	83.5 ± 14.3	< 0.001
SBP (mmHg), Mean ± SD	124.5 ± 11.5	124.1 ± 11.2	125.3 ± 12.0	0.038
DBP (mmHg), Mean ± SD	65.1 ± 9.0	65.7 ± 8.7	64.1 ± 9.4	< 0.001
MBP (mmHg), Mean ± SD	83.7 ± 8.2	84.1 ± 8.0	83.0 ± 8.6	0.009
Respiratory rate (time/min), Mean ± SD	17.5 ± 2.9	17.1 ± 2.7	18.2 ± 3.1	< 0.001
Temperature (°C), Mean ± SD	37.0 ± 0.4	36.9 ± 0.4	37.0 ± 0.5	< 0.001
SpO2 (%), Mean ± SD	97.5 ± 1.7	97.2 ± 1.7	97.8 ± 1.7	< 0.001
**Laboratory**				
Glucose (mg/dL), Median (IQR)	142.3 (127.0, 163.5)	140.6 (125.0, 158.1)	147.0 (130.8, 172.9)	< 0.001
Hematocrit (%), Mean ± SD	33.8 ± 5.7	34.6 ± 5.1	32.4 ± 6.2	< 0.001
Hemoglobin (g/L), Mean ± SD	11.3 ± 2.0	11.6 ± 1.8	10.8 ± 2.1	< 0.001
Platelets (10^9^ /L), Mean ± SD	214.3 ± 80.9	225.4 ± 78.2	196.0 ± 82.0	< 0.001
WBC (10^9^ /L), Median (IQR)	14.2 (11.2, 17.7)	14.1 (11.1, 17.4)	14.4 (11.2, 18.2)	0.181
Aniongap (mmol/L), Mean ± SD	12.9 ± 2.9	13.0 ± 2.8	12.7 ± 3.1	0.087
Bicarbonate (mmol/L), Mean ± SD	22.1 ± 3.1	22.4 ± 3.0	21.8 ± 3.3	< 0.001
BUN (mg/dL), Mean ± SD	16.1 ± 7.3	15.2 ± 6.3	17.7 ± 8.4	< 0.001
Calcium (mg/dL), Mean ± SD	8.3 ± 0.7	8.4 ± 0.6	8.2 ± 0.8	< 0.001
Chloride (mmol/L), Mean ± SD	104.0 ± 4.8	104.3 ± 4.3	103.5 ± 5.5	0.002
Creatinine (mEq/L), Mean ± SD	0.9 ± 0.3	0.8 ± 0.3	1.0 ± 0.4	< 0.001
Sodium (mmol/L), Mean ± SD	141.1 ± 4.3	140.7 ± 3.8	141.7 ± 4.8	< 0.001
Potassium (mmol/L), Mean ± SD	3.8 ± 0.4	3.9 ± 0.4	3.7 ± 0.5	< 0.001
INR, Mean ± SD	1.2 ± 0.4	1.2 ± 0.3	1.3 ± 0.6	< 0.001
PT(seconds), Mean ± SD	13.5 ± 5.6	12.8 ± 2.7	14.6 ± 8.3	< 0.001
PTT (seconds), Mean ± SD	28.8 ± 12.5	27.7 ± 10.6	30.5 ± 14.9	< 0.001
**Comorbidities**				
Myocardial infarct, n (%)	66 (4.1)	25 (2.5)	41 (6.8)	< 0.001
Congestive heart failure, n (%)	69 (4.3)	24 (2.4)	45 (7.4)	< 0.001
Peripheral vascula disease, n (%)	50 (3.1)	29 (2.9)	21 (3.5)	0.536
dementia, n (%)	26 (1.6)	11 (1.1)	15 (2.5)	0.035
Cerebrovascular disease, n (%)	395 (24.6)	200 (20)	195 (32.1)	< 0.001
Chronic pulmonary disease, n (%)	181 (11.3)	106 (10.6)	75 (12.4)	0.287
Rheumatic disease, n (%)	30 (1.9)	15 (1.5)	15 (2.5)	0.165
Peptic ulcer disease, n (%)	5 (0.3)	1 (0.1)	4 (0.7)	0.071
Mild liver disease, n (%)	51 (3.2)	26 (2.6)	25 (4.1)	0.094
Diabetes without chronic complications, n (%)	209 (13.0)	96 (9.6)	113 (18.6)	< 0.001
Diabetes with chronic complications, n (%)	37 (2.3)	17 (1.7)	20 (3.3)	0.039
Paraplegia, n (%)	188 (11.7)	97 (9.7)	91 (15)	0.001
Malignant cancer, n (%)	385 (24.0)	288 (28.9)	97 (16)	< 0.001
Severe liver disease, n (%)	10 (0.6)	2 (0.2)	8 (1.3)	0.008
Metastatic solid tumor, n (%)	230 (14.3)	172 (17.2)	58 (9.6)	< 0.001
AIDS, n (%)	5 (0.3)	4 (0.4)	1 (0.2)	0.656
**Severity scores**				
Charlson comorbidity index, Median (IQR)	4.0 (2.0, 6.0)	4.0 (2.0, 6.0)	4.0 (3.0, 6.0)	0.081
APSIII, Mean ± SD	36.0 ± 18.0	30.8 ± 13.6	44.4 ± 21.0	< 0.001
SAPSII, Mean ± SD	29.6 ± 11.0	27.3 ± 10.2	33.4 ± 11.4	< 0.001
OASIS, Mean ± SD	27.6 ± 9.3	24.4 ± 7.8	32.9 ± 9.1	< 0.001
**Perioperative exposures**				
CRRT, n (%)	4 (0.2)	0 (0)	4 (0.7)	0.02
Mechanical ventilation, n (%)	147 (9.2)	47 (4.7)	100 (16.5)	< 0.001
Mannitol, n (%)	50 (3.1)	26 (2.6)	24 (4)	0.132
Transfusion, n (%)	177 (11.0)	60 (6)	117 (19.3)	< 0.001
Hypotension_24h, n (%)	643 (40.1)	376 (37.7)	267 (44)	0.012
Antiplatelet, n (%)	63 (3.9)	39 (3.9)	24 (4)	0.963
**Surgical procedure type**				< 0.001
Tumor / Resection, n (%)	981 (61.1)	710 (71.1)	271 (44.6)	
Trauma / Decompression, n (%)	86 (5.4)	42 (4.2)	44 (7.2)	
Other / CSF diversion, n (%)	538 (33.5)	246 (24.6)	292 (48.1)	
**Length of stay**				
LOS hospital (d), Median (IQR)	6.8 (3.8, 12.9)	5.6 (3.5, 9.7)	9.4 (5.7, 19.1)	< 0.001
LOS ICU (d), Median (IQR)	2.6 (1.6, 5.0)	2.0 (1.3, 3.2)	4.1 (2.4, 8.2)	< 0.001

SPO2, saturation of percutaneous oxygen; SBP, systolic blood pressure; DBP, diastolic blood pressure; MBP, mean blood pressure; WBC, white blood cell; BUN, blood urea nitrogen; INR, international normalized ratio; PT, prothrombin time; PTT, partial thromboplastin time; APS III, acute physiology score III; SAPS II, simplified acute physiology score II; OASIS, Oxford acute severity of illness score; LOS hospital, length of stay in the hospital; LOS ICU, length of stay in the ICU.

#### 3.1.2. Univariate logistic regression analysis and correlational analysis.

**Fig 2:**
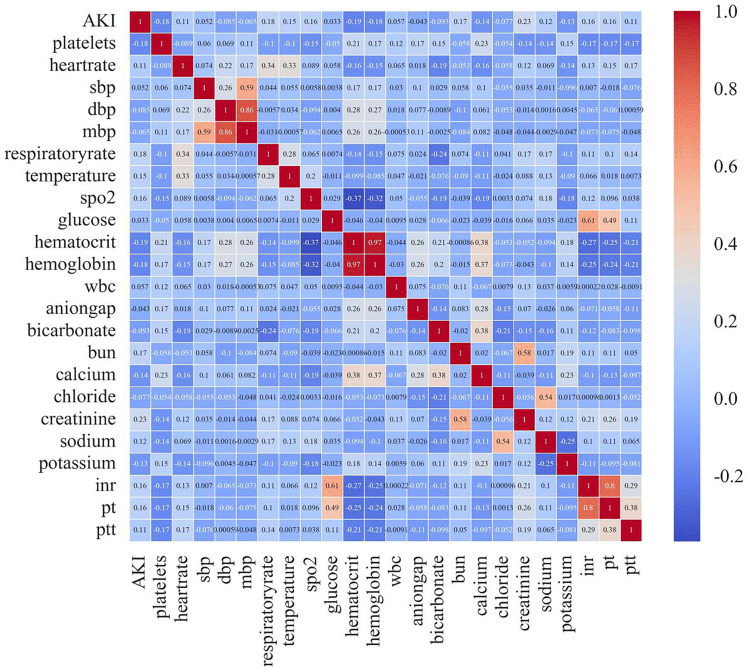
Multivariate correlation analysis.

As shown in **Table 2**, univariate logistic regression analysis revealed a positive association between the incidence of acute kidney injury (AKI) and factors such as gender, age, heart rate, SBP, body temperature, respiratory rate, BUN, serum creatinine levels, and several scoring systems. Conversely, a negative association was observed with variables such as DBP, MAP, hematocrit, hemoglobin concentration, anion gap, bicarbonate levels, as well as serum calcium, chloride, and potassium concentrations. The heatmap from the correlation analysis (**Fig 2**) further indicates a notable relationship between platelet counts and the occurrence of AKI (correlation coefficient: – 0.176, P < 0.001).

**Table 2:** Univariate logistic analysis of platelet levels and the incidence of AKI within 7 days of ICU admission.

Variable	HR (95 CI)	*p*-value
Platelets	0.99 (0.99 ~ 1)	<0.001
Gender (Male)	1.47 (1.2 ~ 1.81)	<0.001
Age	1.01 (1.01 ~ 1.02)	<0.001
Race (African American)	1.27 (0.88 ~ 1.84)	0.205
Race (Asian)	0.95 (0.55 ~ 1.64)	0.855
Race (Other)	1.43 (1.12 ~ 1.83)	0.004
Heart rate	1.02 (1.01 ~ 1.02)	<0.001
SBP	1.01 (1 ~ 1.02)	0.038
DBP	0.98 (0.97 ~ 0.99)	0.001
MBP	0.98 (0.97 ~ 1)	0.009
Respiratory rate	1.14 (1.1 ~ 1.18)	<0.001
Temperature	2.06 (1.62 ~ 2.63)	<0.001
SpO_2_	1.22 (1.14 ~ 1.29)	<0.001
Glucose	1.01 (1 ~ 1.01)	<0.001
Hematocrit	0.93 (0.92 ~ 0.95)	<0.001
Hemoglobin	0.82 (0.78 ~ 0.87)	<0.001
WBC	1.02 (1 ~ 1.03)	0.039
Aniongap	0.97 (0.94 ~ 1)	0.087
Bicarbonate	0.94 (0.91 ~ 0.97)	<0.001
Bun	1.05 (1.03 ~ 1.07)	<0.001
Calcium	0.66 (0.56 ~ 0.76)	<0.001
Chloride	0.97 (0.95 ~ 0.99)	0.002
Creatinine	5.5 (3.71 ~ 8.14)	<0.001
Sodium	1.06 (1.03 ~ 1.09)	<0.001
Potassium	0.55 (0.43 ~ 0.69)	<0.001
INR	4.34 (2.79 ~ 6.77)	<0.001
PT	1.17 (1.12 ~ 1.22)	<0.001
PTT	1.02 (1.01 ~ 1.03)	<0.001
Myocardial infarct	2.82 (1.7 ~ 4.69)	<0.001
Congestive heart failure	3.25 (1.96 ~ 5.39)	<0.001
Peripheral vascular disease	1.2 (0.68 ~ 2.12)	0.536
Dementia	2.27 (1.04 ~ 4.98)	0.04
Cerebrovascular disease	1.89 (1.5 ~ 2.38)	<0.001
Chronic pulmonary disease	1.19 (0.87 ~ 1.62)	0.287
Rheumatic disease	1.66 (0.81 ~ 3.42)	0.169
Peptic ulcer disease	6.61 (0.74 ~ 59.31)	0.091
Mild liver disease	1.61 (0.92 ~ 2.81)	0.096
Diabetes without chronic complications	2.15 (1.6 ~ 2.88)	<0.001
Diabetes with chronic complications	1.97 (1.02 ~ 3.78)	0.043
Paraplegia	1.64 (1.21 ~ 2.22)	0.002
Malignant cancer	0.47 (0.36 ~ 0.61)	<0.001
Severe liver disease	6.65 (1.41 ~ 31.43)	0.017
Metastatic solid tumor	0.51 (0.37 ~ 0.7)	<0.001
AIDS	0.41 (0.05 ~ 3.68)	0.426
Charlson comorbidity index	1.02 (0.99 ~ 1.05)	0.268
APSIII	1.05 (1.04 ~ 1.05)	<0.001
SAPSII	1.05 (1.04 ~ 1.06)	<0.001
OASIS	1.12 (1.1 ~ 1.13)	<0.001
Mechanical ventilation	3.99 (2.78 ~ 5.74)	<0.001
LOS hospital	1.04 (1.03 ~ 1.05)	<0.001
LOS ICU	1.18 (1.15 ~ 1.22)	<0.001

SPO2, saturation of percutaneous oxygen; SBP, systolic blood pressure; DBP, diastolic blood pressure; MBP, mean blood pressure; WBC, white blood cell; BUN, blood urea nitrogen; INR, international normalized ratio; PT, prothrombin time; PTT, partial thromboplastin time; APS III, acute physiology score III; SAPS II, simplified acute physiology score II; OASIS, Oxford acute severity of illness score; LOS hospital, length of stay in the hospital; LOS ICU, length of stay in the ICU.

#### 3.1.3. The impact of platelet levels on AKI.

**Table 3** displays the results of a multivariate logistic regression analysis, using the lowest tertile of platelet counts as the reference category. The data reveal a statistically significant inverse association between elevated platelet levels and the risk of AKI in neurosurgical patients admitted to the ICU. After controlling for potential confounding variables, individuals in the second tertile (179 < PLT ≤ 234) and the third tertile (PLT > 234) demonstrated a decreased likelihood of developing AKI compared to those in the lowest tertile (PLT < 179). These findings imply that neurosurgical ICU patients with lower platelet counts are more susceptible to developing AKI within the first seven days of admission.

**Table 3:** Analysis of the Association between Platelet Levels and the Incidence of Acute Kidney Injury (AKI) within 7 Days after ICU Admission among Neurosurgical Patients in a Multivariate Regression Model.

Variable	Model 1		Model 2		Model 3		Model 4	
	HR (95%CI)	*P*-value	HR (95%CI)	*P*-value	HR (95%CI)	*P*-value	HR (95%CI)	*P*-value
PLT tertials								
<179	1(Reference)		1(Reference)		1(Reference)		1(Reference)	
179-234	0.57 (0.45 ~ 0.73)	<0.001	0.45 (0.35 ~ 0.58)	<0.001	0.73 (0.56 ~ 0.94)	0.017	0.76 (0.58 ~ 0.99)	0.045
>234	0.42 (0.33 ~ 0.54)	<0.001	0.67 (0.59 ~ 0.76)	<0.001	0.57 (0.43 ~ 0.74)	<0.001	0.62 (0.47 ~ 0.82)	0.001
*P* for trend		<0.001		<0.001	0.75 (0.66 ~ 0.86)	<0.001	0.79 (0.68 ~ 0.91)	0.001

Model 1: No adjusted.

Model 2: Adjusted for age and gender.

Model 3: Adjusted for age, gender, procedure type, transfusion, mannito, hypotension, antiplatelet.

Model 4: Adjusted for model 3 plus Bun, creatinine, congestive heart failure, CRRT and mechanical ventilation.

**Fig 3:**
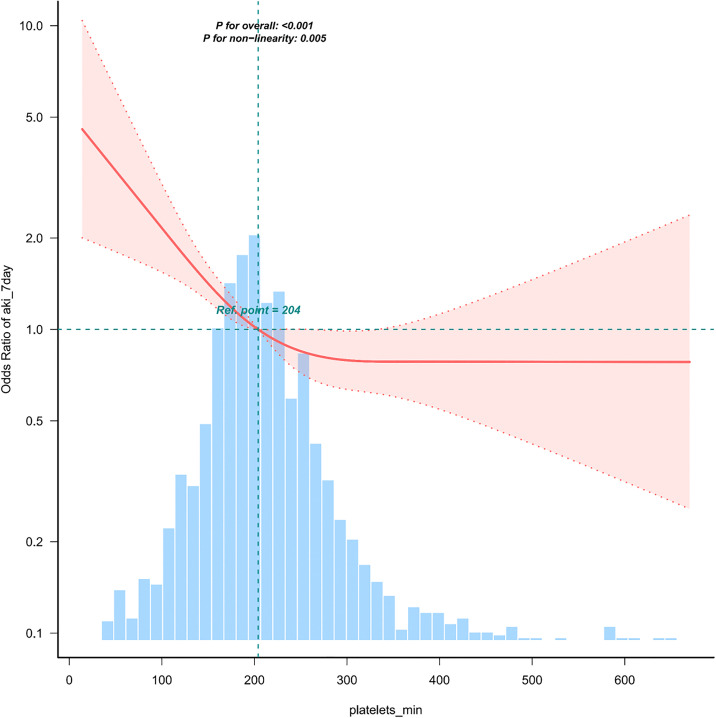
The association between platelet levels and the risk of AKI within 7 days after admission to the ICU in neurosurgical patients.

The restricted cubic spline analysis shown in **Fig 3** indicates a significant nonlinear relationship between platelet levels and the risk of AKI in neurosurgical patients after admission to the ICU (P < 0.01). Specifically, lower platelet levels are associated with a higher risk of AKI. When PLT < 204 × 10⁹/L, the risk of AKI significantly decreases as platelet levels increase; however, when PLT > 204 × 10⁹/L, although the risk of AKI still decreases with increasing platelet levels, the rate of decline is significantly slower.

#### 3.1.4. Subgroup analysis.

**Fig 4:**
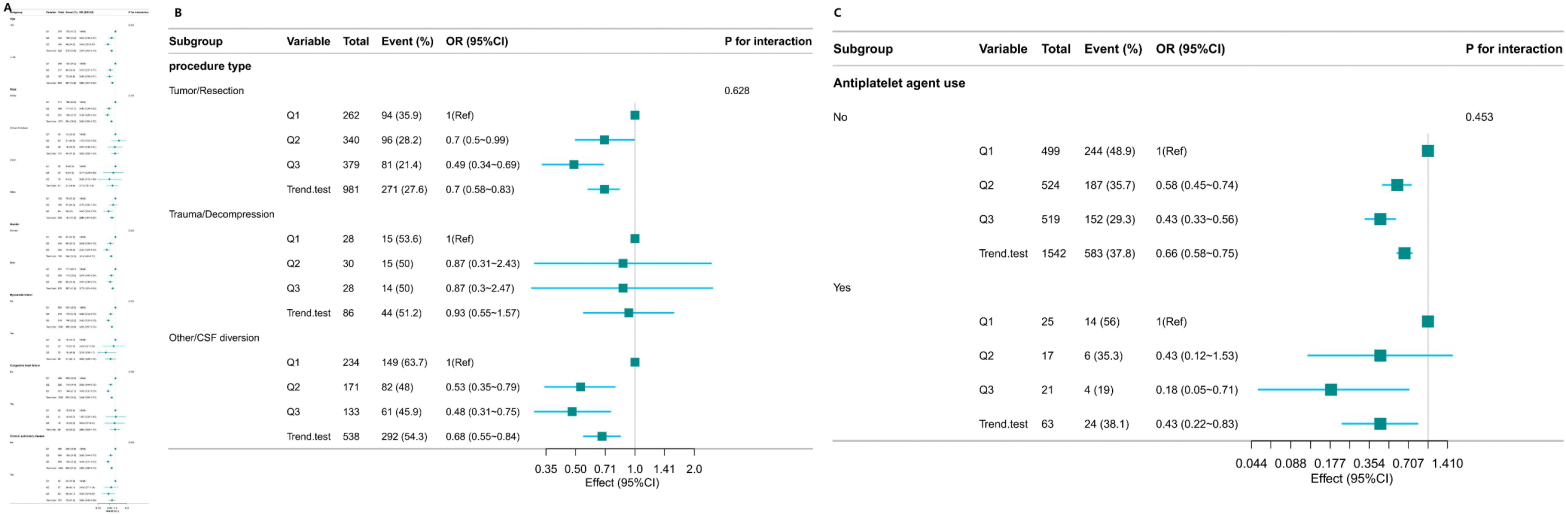
(A-C). The relationship between platelet levels and the occurrence of AKI within 7 days after admission to the ICU in neurosurgical patients in subgroup analysis.

**Fig 4A** shows the relationship between platelet levels and the occurrence of AKI within 7 days after admission to the ICU in neurosurgical patients evaluated through subgroup analysis. After stratification analysis based on factors such as age, race, gender, history of myocardial infarction, history of congestive heart failure, and chronic obstructive pulmonary disease, no significant interaction was found among the subgroups (P-Values for interaction terms were all greater than 0.05). Subgroup analyses (**Fig 4B**) stratified by surgical type (Tumor/Resection, Trauma/Decompression, and Other/Cerebrospinal Fluid Diversion) demonstrated consistent associations between platelet tertiles and AKI across subgroups, with no statistically significant interaction detected (P for interaction = 0.628). **Fig 4C** presents subgroup analysis results stratified by the use of early antiplatelet agents (aspirin, clopidogrel, or ticagrelor) within 24 hours of ICU admission. Among patients without early antiplatelet exposure, lower platelet count categories were associated with a stepwise increase in AKI risk. A comparable directional association was observed in patients who received early antiplatelet therapy; however, the confidence intervals were wider due to a smaller sample size. Importantly, no statistically significant interaction was detected between platelet count categories and early antiplatelet agent use with respect to AKI risk (interaction P > 0.05).

#### 3.1.5. Sensitivity analysis.

In sensitivity analyses with the first platelet count post-ICU admission and the mean platelet count within the first 24 hours of ICU admission as exposure variables, analogous nonlinear associations between platelet levels and AKI risk were observed, characterized by comparable nadir risk points and consistent overall trends (**S1A–B Fig**).

### 3.2. Analysis of the long-term prognosis (1-year mortality rate)

#### 3.2.1. Bounded cubic spline function.

**Fig 5:**
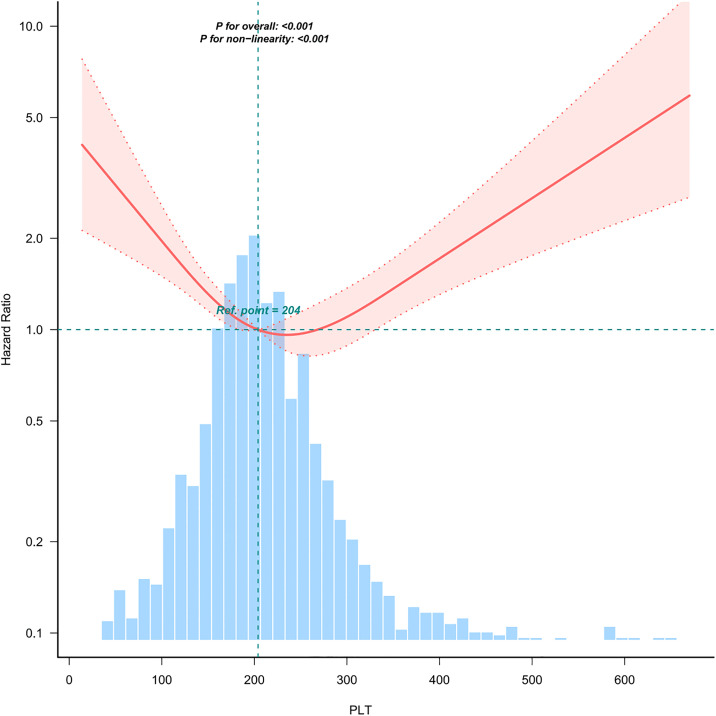
The association between platelet levels within 7 days after admission to the ICU and one-year mortality risk in neurosurgical patients.

**Fig 5** illustrates the association between 1-year mortality and platelet counts measured on the first day of hospital admission in patients who received surgical interventions. The findings indicate that both abnormally low and high platelet levels were linked to an increased risk of death within one year. This finding indicates a J-shaped curve association between platelet levels and the 1-year mortality rate in this patient cohort (P < 0.001). Based on the restricted cubic spline analysis, within our tertile stratification, the platelet level range corresponding to the lowest 1-year mortality risk was 179–234 (10^9^/L). When the platelet level was lower than 179, the risk increased steeply. Beyond 234, although the risk continued to rise, the trend was more gradual, presenting a plateau effect. This phenomenon elucidates the intricate and non-linear relationship characteristics between platelet levels and the one-year mortality rate among these patients.

#### 3.2.2. Multivariable Cox regression analysis.

**Table 4** displays the unadjusted and adjusted estimates concerning the association between platelet levels within 24 hours subsequent to admission to the ICU and the one-year mortality rate among neurosurgical patients, as derived from the Cox regression model. When considering SpO₂ as a categorical variable, using the second platelet-level subgroup (Q2, 179 < PLT ≤ 234) as the reference group, following comprehensive adjustment, the one-year mortality risk in the low-platelet-level subgroup (Q1, PLT ≤ 179) was notably elevated (HR1: 1.45, [95% CI: 1.11–1.9], P = 0.007). In the high-platelet-level subgroup (Q3, PLT > 234), although the one-year mortality risk was indicated to increase, the difference was not statistically significant (HR2: 1.15, [95% CI: 0.87–1.52], P > 0.05).

**Table 4:** The hazard ratios and 95% confidence intervals of platelets in relation to the one – year mortality rate.

Variable	Model 1		Model 2		Model 3	
	HR (95%CI)	*P*-value	HR (95%CI)	*P*-value	HR (95%CI)	*P*-value
PLT tertials						
<179	1.53 (1.17 ~ 2)	0.002	1.4 (1.07 ~ 1.83)	0.014	1.45 (1.11 ~ 1.9)	0.007
179-234	1(Reference)		1(Reference)		1(Reference)	
>234	0.86 (0.75 ~ 0.98)	0.316	1.17 (0.89 ~ 1.55)	0.256	1.15 (0.87 ~ 1.52)	0.329

Model 1: No adjusted.

Model 2: Adjusted for age and gender.

Model 3: Adjusted for age, gender, temperature, BUN, peripheral vascular disease, and charlson comorbidity index.

#### 3.2.3. Cumulative mortality curve analysis.

**Fig 6:**
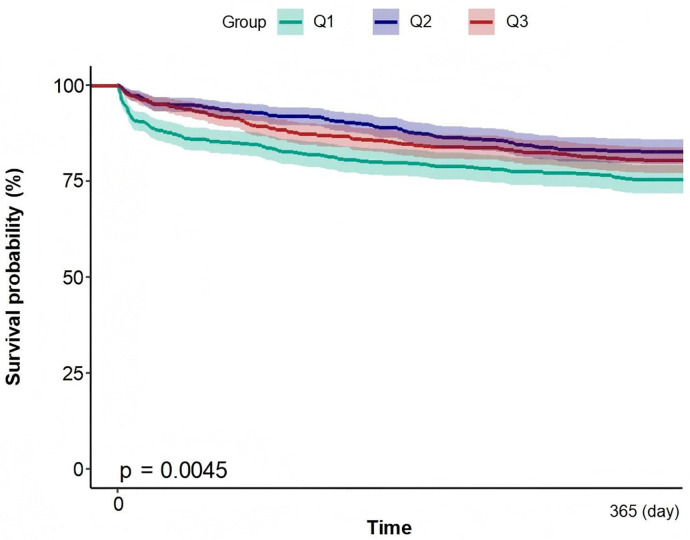
Cumulative mortality curves within 365 days after admission for patients in different platelet level groups.

By precisely calculating the daily cumulative mortality rate of patients in three different platelet level subgroups within 365 days after admission, Fig 6 clearly shows a stratified mortality trend: the normal platelet level group (Q2, 179 < PLT ≤ 234), the high platelet level group (Q3, PLT > 234), and the low platelet level group (Q1, PLT ≤ 179). Notably, this observed trend is highly consistent with the results of the Cox multivariate regression analysis.

#### 3.2.4. Subgroup analysis.

**Fig 7:**
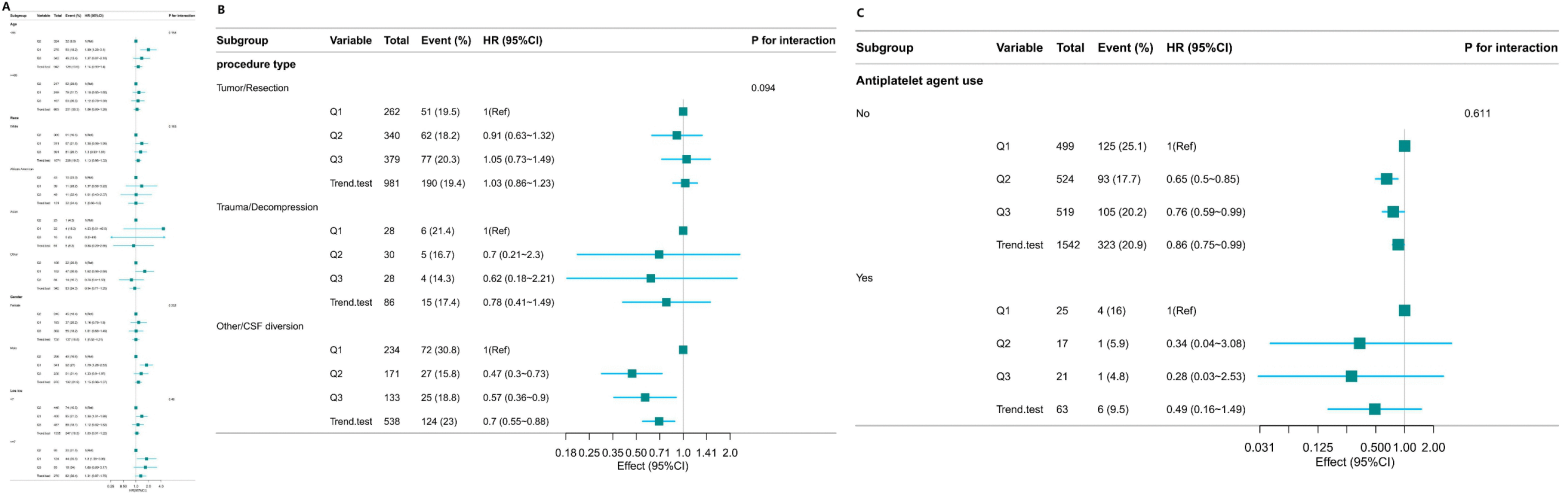
(A-C). Forest Plot of Subgroup Analysis on the Association between Platelet Levels and One – year Mortality.

During the subgroup analysis (as shown in Fig 7A), no statistically significant interactions were detected between platelet counts and factors such as age, ethnicity, sex, or duration of intensive care unit admission. All corresponding P-Values exceeded 0.05. In the surgical type-stratified subgroup analysis for 1-year mortality (Fig 7B), the interaction test did not achieve statistical significance (P for interaction = 0.094), indicating insufficient evidence for a modifying effect of surgical type on the association. Fig 7C presents subgroup analyses stratified by early antiplatelet agent use. Among patients without early antiplatelet exposure, lower platelet count categories were associated with an elevated 1-year mortality risk. A consistent trend was observed in patients who received antiplatelet agents within 24 hours of ICU admission. No statistically significant interaction was detected between platelet levels and early antiplatelet agent use with respect to 1-year mortality (interaction P > 0.05), indicating that early antiplatelet therapy did not modify the association between platelet count and long-term survival.

#### 3.2.5. Sensitivity analysis.

In sensitivity analyses with the first platelet count post-ICU admission and the mean platelet count within the first 24 hours of ICU admission as exposure variables, the nonlinear association between platelet levels and 1-year mortality remained statistically significant, with comparable curve morphology and nadir risk points to the primary analysis (**S2A–B Fig**).

### 3.3. External validation

**S3 Fig** presents the patient selection process for the external validation cohort, which applied identical inclusion and exclusion criteria as the derivation cohort. **S4 Fig** shows the nonlinear association between the lowest platelet count within 24 hours after ICU admission and **(A)** the risk of AKI within 7 days (using a logistic regression model) and **(B)** 1-year all-cause mortality (using a Cox proportional hazards model) in the MIMIC-III cohort as demonstrated by restricted cubic spline analysis. Both models revealed a highly significant overall and nonlinear association between platelet levels and outcomes (overall P < 0.001; nonlinear P < 0.001).

## 4. Discussion

During the early perioperative period of critically ill neurosurgical patients, platelet counts exhibit marked dynamic changes, which are primarily driven by acute pathophysiological processes. In the immediate postoperative phase and the initial stage following ICU admission, massive fluid resuscitation and administration of vasoactive agents may induce hemodilutional thrombocytopenia. Concurrently, factors including surgical trauma, vascular endothelial injury, active hemorrhage, infection, and thrombosis can further augment the acute consumption of platelets [17]. Prior research has demonstrated that platelet counts typically reach the nadir within 24–48 hours postoperatively, reflecting the combined effects of acute injury and perioperative physiological stress [18]. Beyond 72 hours, however, platelet levels may gradually increase due to compensatory bone marrow hyperplasia, thereby masking the severity of early-stage injury [17]. Hence, in the present study, the minimum platelet count within 24 hours after ICU admission was selected to capture the critical time window of perioperative acute hemodilution and platelet consumption, thus more authentically reflecting the overall burden of early postoperative microcirculatory dysfunction and coagulation system derangement. Through multivariable logistic regression analyses, our study demonstrated that, among neurosurgical patients admitted to the ICU, lower platelet levels within the first 24 hours were associated with a higher risk of AKI within 7 days. In addition, this retrospective cohort study revealed a significant association between early platelet levels and 1-year mortality. Restricted cubic spline analyses further illustrated a J-shaped non-linear relationship between platelet count and long-term mortality, indicating that both markedly low and high platelet levels were associated with increased mortality risk, with the association being more pronounced at lower platelet levels.

In neurosurgical patients, AKI and thrombocytopenia frequently co-occur and exert notable adverse impacts on clinical outcomes. The incidence of AKI among patients after neurosurgical operations ranges from 10% to 14% [19]. Thrombocytopenia is especially prevalent in children undergoing neurosurgical procedures. Specifically, in patients receiving intravenous valproic acid (VPA) treatment, its incidence is affected by the baseline platelet count. Those with a lower baseline platelet count face a higher risk [20]. Post-operative thrombocytopenia is closely associated with a variety of adverse prognoses. These include an elevated mortality rate, an extended length of stay in the ICU, as well as the occurrence of complications such as AKI, stroke, limb ischemia, and mesenteric ischemia [21,22]. In patients with traumatic brain injury (TBI), around 10% develop early-onset thrombocytopenia during their ICU admission, and this is an independent risk factor for death in patients with moderate to severe TBI [23]. Moreover, late-onset thrombocytopenia is strongly associated with a significantly shortened survival time [22]. AKI itself may act as one of the contributing factors to thrombocytopenia. In individuals with impaired renal function, the incidence of severe thrombocytopenia is higher [24].

The existing literature has indicated that platelet count is associated with AKI and mortality in ICU patients. However, the majority of studies have concentrated on non-surgical populations or specific disease groups (such as stroke or respiratory failure), lacking a specific analysis for patients following neurosurgical procedures [25–27]. This study represents the first attempt to validate the non-linear effect of platelet count on the risk of AKI within a post-neurosurgical cohort, thus filling a theoretical void in this domain. Previous research has shown that in patients with end-stage renal disease (ESRD), a platelet count of around 222 × 10⁹/L is associated with the lowest mortality rate [26]. In contrast, this study extends the generalizability of our findings to the post-neurosurgical population, with a focus on highlighting population-specific risk patterns rather than establishing universally applicable therapeutic thresholds. Furthermore, this study comprehensively employs logistic regression and the Cox proportional hazards model, enabling a dual validation of both the short-term AKI risk and long-term prognosis. Compared to studies that solely focus on a single endpoint, this approach offers more clinically instructive insights. Importantly, the observed non-linear associations were consistently replicated in an independent external cohort derived from the MIMIC-III database.

In neurosurgical patients, thrombocytopenia may mirror the severity of surgical trauma and the systemic inflammatory response [28]. Neurosurgical operations, such as the resection of brain tumors or surgeries for traumatic brain injury, are frequently accompanied by pronounced inflammatory responses, including the elevation of interleukin-6 (IL-6), tumor necrosis factor-α (TNF-α), etc. The levels of these inflammatory factors are significantly correlated with coagulation parameters, disseminated intravascular coagulation (DIC), and AKI [29,30]. Simultaneously, inflammatory factors can promote platelet consumption by activating platelets, thereby resulting in thrombocytopenia [31]. Numerous studies have indicated that postoperative thrombocytopenia is a crucial predictive indicator of multiple organ dysfunction syndrome (MODS) in patients. It is independently associated with an increased risk of mortality, AKI, and other organ complications [21,32]. Accordingly, thrombocytopenia is more likely to serve as a marker of disease severity rather than a direct causal factor.

More significantly, thrombocytopenia may exacerbate renal injury via multiple mechanisms: (1) Coagulation-fibrinolysis imbalance: Thrombocytopenia often coincides with the early alterations of DIC. The formation of microthrombi can cause microcirculatory disorders in the kidneys [31,33,34]. (2) Endothelial injury and hypoperfusion: Inflammatory responses, such as the activation of endothelial cells by TNF-α, can weaken the repair ability of endothelial cells and intensify ischemic tubular injury in the kidneys [35,36]. (3) Uncontrolled inflammatory response: Platelets play a role in immunoregulation. A decrease in their number may lead to the excessive formation of neutrophil extracellular traps (NETs), thereby exacerbating renal inflammatory injury through the immunothrombotic mechanism [37,38]. Collectively, these mechanisms contribute to the development of AKI in this group of patients.

Post-operative thrombocytopenia can remarkably augment the risk of bleeding events. In this group of patients, the incidence of major hemorrhage (> 400 ml) is relatively high, and platelet transfusion is frequently necessary [39]. Persistent bleeding not only gives rise to hemorrhagic shock and organ hypoperfusion but may also indirectly prolong the length of stay in the ICU due to complications associated with transfusion, such as infections and immune responses [40]. It is worth noting that a dynamic decline in platelet count is more strongly correlated with 30-day mortality. This indicates that the progressive deterioration of the coagulation system may exacerbate organ damage through a vicious cycle of bleeding and transfusion, ultimately influencing the 1-year survival rate [41]. Furthermore, thrombocytopenia often serves as a biological indicator of severe inflammatory responses. Research has revealed that patients in the low-platelet-count group exhibit significantly elevated inflammatory markers upon admission. This may be attributed to the extensive consumption of platelets after their over-activation by inflammatory factors [42]. Meanwhile, during the process of microthrombus formation, the aggregation of platelets and leukocytes exacerbates endothelial injury, and platelet-derived microparticles further promote the formation of NETs [37,43]. This inflammatory micro-environment has a high propensity to trigger postoperative sepsis. A decrease in platelet count by more than 25% has been proven to be independently associated with the 30-day mortality of sepsis patients [44]. Beyond being a marker of injury, thrombocytopenia may directly contribute to the exacerbation of organ failure. A reduction in platelet-derived growth factors (PDGF/VEGF) can impede endothelial repair, thereby intensifying the chronic progression following AKI [39,45]. Studies have shown that in patients after cardiac surgery, a dynamic decline in platelet count is significantly associated with cardiogenic shock. Myocardial microcirculation disorders may impact long-term prognosis through persistent cardiac dysfunction [41]. Additionally, patients with elevated lactate levels are more likely to experience decompensation of liver and kidney function, suggesting that thrombocytopenia may expand the scope of organ damage via hypoxia-reperfusion injury [45,46]. These complex interactions among multiple systems ultimately increase the risk of patient mortality.

Our study suggests that platelet levels may be associated with the risk of AKI and long-term prognosis in patients admitted to neurosurgical intensive care units. Early dynamic monitoring of platelet trajectories, combined with comprehensive assessment using the Acute Physiology and Chronic Health Evaluation II (APACHE-II) score, may enhance AKI risk stratification and clinical early warning systems. Nevertheless, prospective studies are warranted to validate whether interventions targeting platelet abnormalities—such as platelet transfusion or pharmacological therapy—can reduce the incidence of AKI and improve long-term outcomes.

### 4.1. Limitations and future prospects

Meanwhile, this study has several limitations. Firstly, given its retrospective design, it is challenging to fully rule out the confounding effects of underlying diseases, medications, and other factors. This may introduce certain biases into the results. Secondly, platelet count does not comprehensively capture the functional status of platelets, which may exert a critical role in the pathophysiological mechanisms underlying AKI. Owing to the retrospective design of this study and the absence of systematic platelet function assay data in the MIMIC-IV database, we were unable to evaluate platelet aggregation capacity or global functional activity. Thirdly,future multi-center investigations are essential to validate and generalize these findings. For subsequent research, prospective cohort studies are recommended. These should be complemented by molecular mechanism analyses to identify neurosurgery-specific biomarkers. Additionally, optimizing real-time monitoring tools will be crucial for implementing personalized intervention strategies. This approach will contribute to a more comprehensive understanding of the subject and potentially improve patient outcomes.

## 5. Conclusion

In critically ill neurosurgical patients, platelet counts measured early after admission to the ICU demonstrated a significant non-linear association with both the risk of AKI within 7 days post-surgery and 1-year all-cause mortality. Specifically, the risk of AKI increased significantly when platelet counts fell below approximately 204 × 10⁹/L. Additionally, both extremely low and extremely high platelet counts were associated with elevated mortality risk, with a stronger correlation observed between marked thrombocytopenia and adverse long-term outcomes. After multivariate adjustment, patients with platelet levels in the range of 179–234 × 10⁹/L exhibited a lower AKI risk and higher survival probability. These findings suggest that early post-admission platelet counts may serve as a readily accessible biomarker for risk stratification and early identification of postoperative AKI and long-term prognosis. However, given the retrospective observational design of this study, the aforementioned range should be interpreted as a statistically associated range corresponding to the lowest risk, rather than a definitive therapeutic target for clinical intervention. Further prospective studies are warranted to validate the causal relationship and optimize clinical application strategies.

## Supporting information

S1 Fig Sensitivity Analysis of Platelet Levels Within 7 Days After ICU Admission and AKI Risk in Neurosurgical Patients: (A) First Platelet Measurement Following ICU Admission and AKI Risk; (B) Mean Platelet Value Within 24 Hours After ICU Admission and AKI Risk. (TIF)

S2 Fig Sensitivity Analysis of Platelet Levels Following ICU Admission and 1-Year Mortality Risk in Neurosurgical Patients: (A) First Platelet Measurement After ICU Admission and 1-Year Mortality Risk; (B) Mean Platelet Value Within 24 Hours After ICU Admission and 1-Year Mortality Risk. (TIF)

S3 Fig Flowchart of Inclusion and Exclusion Criteria for the External Validation Cohort. (TIF)

S4 Fig External Validation of Restricted Cubic Splines: (A) Platelet Count and Risk of Acute Kidney Injury (AKI) Within 7 Days; (B) Platelet Count and 1-Year All-Cause Mortality. (TIF)
